# The reproducibility of acquiring three dimensional gait and plantar pressure data using established protocols in participants with and without type 2 diabetes and foot ulcers

**DOI:** 10.1186/s13047-016-0135-8

**Published:** 2016-01-29

**Authors:** Malindu Fernando, Robert G Crowther, Margaret Cunningham, Peter A Lazzarini, Kunwarjit S Sangla, Petra Buttner, Jonathan Golledge

**Affiliations:** Vascular Biology Unit, Queensland Research Centre for Peripheral Vascular Disease, College of Medicine and Dentistry, James Cook University, Townsville, QLD 4811 Australia; Institute of Sports and Exercise Science, Gait analysis Laboratory, James Cook University, Townsville, Australia; Department of Diabetes and Endocrinology, The Townsville Hospital, Townsville, QLD Australia; Allied Health Research Collaborative, Metro North Hospital & Health Service, Queensland Health, Brisbane, Australia; School of Clinical Sciences, Queensland University of Technology, Brisbane, Australia; Department of Vascular and Endovascular Surgery, The Townsville Hospital, Queensland, Australia; Department of Health Science, University of Stirling, Stirling, Scotland United Kingdom; Tropical Health Solutions Pty Ltd, Townsville, QLD Australia; Sport and Exercise, School of Health and Wellbeing, University of Southern Queensland, Queensland, Australia; Centre for Chronic Disease Prevention, College of Public Health, Medical and Veterinary Sciences, James Cook University, Townsville, Australia

**Keywords:** Diabetic foot, Reproducibility of results, Gait, Locomotion, Plantar pressure, Foot ulcer

## Abstract

**Background:**

Several prospective studies have suggested that gait and plantar pressure abnormalities secondary to diabetic peripheral neuropathy contributes to foot ulceration. There are many different methods by which gait and plantar pressures are assessed and currently there is no agreed standardised approach. This study aimed to describe the methods and reproducibility of three-dimensional gait and plantar pressure assessments in a small subset of participants using pre-existing protocols.

**Methods:**

Fourteen participants were conveniently sampled prior to a planned longitudinal study; four patients with diabetes and plantar foot ulcers, five patients with diabetes but no foot ulcers and five healthy controls. The repeatability of measuring key biomechanical data was assessed including the identification of 16 key anatomical landmarks, the measurement of seven leg dimensions, the processing of 22 three-dimensional gait parameters and the analysis of four different plantar pressures measures at 20 foot regions.

**Results:**

The mean inter-observer differences were within the pre-defined acceptable level (<7 mm) for 100 % (16 of 16) of key anatomical landmarks measured for gait analysis. The intra-observer assessment concordance correlation coefficients were > 0.9 for 100 % (7 of 7) of leg dimensions. The coefficients of variations (CVs) were within the pre-defined acceptable level (<10 %) for 100 % (22 of 22) of gait parameters. The CVs were within the pre-defined acceptable level (<30 %) for 95 % (19 of 20) of the contact area measures, 85 % (17 of 20) of mean plantar pressures, 70 % (14 of 20) of pressure time integrals and 55 % (11 of 20) of maximum sensor plantar pressure measures.

**Conclusion:**

Overall, the findings of this study suggest that important gait and plantar pressure measurements can be reliably acquired. Nearly all measures contributing to three-dimensional gait parameter assessments were within predefined acceptable limits. Most plantar pressure measurements were also within predefined acceptable limits; however, reproducibility was not as good for assessment of the maximum sensor pressure. To our knowledge, this is the first study to investigate the reproducibility of several biomechanical methods in a heterogeneous cohort.

**Electronic supplementary material:**

The online version of this article (doi:10.1186/s13047-016-0135-8) contains supplementary material, which is available to authorized users.

## Background

It is well established that biomechanical abnormalities secondary to diabetic peripheral neuropathy (DPN) contribute to the formation of diabetic foot ulcers (DFUs) [1–4]. There is limited understanding however regarding how such biomechanical factors influence foot ulcer healing [4–6]. Studies examining the biomechanical factors influencing foot ulcer healing need to perform repeated assessments over time in the same patients [5]. A prerequisite for such studies are reproducible methods [7].

Three dimensional gait and plantar pressure analyses are considered important in assessing the biomechanical characteristics of the foot [4, 5, 8, 9]. There are many different methods by which these analyses have been performed and currently there is no agreed standardised approach [7, 10, 11]. The comparison of results within individuals and between different participants can therefore be difficult [4]. There is a need to better describe the reproducibility of methods used to acquire gait and plantar pressure data and to define ways to minimise measurement error [7, 11–13]. This is especially important prior to interpreting results of studies in which gait is being assessed repeatedly over time, since measurement error will need to be taken into account [7].

This study aimed to describe the methods and the reproducibility of measurements performed during three dimensional gait and plantar pressure assessment. A small group of participants who had diabetes with and without foot ulcers and healthy controls were examined.

## Methods

### Study design and participants

Fourteen participants were conveniently selected from a larger group of people enrolled in a longitudinal study [14]. Participants were selected on the basis of their availability to attend five extra visits required for the assessment of reproducibility. Four participants with type 2 diabetes and active plantar foot ulcers (DFU group) and five participants with type 2 diabetes without active foot ulcers (DMC group) were recruited from The Townsville Hospital, Queensland, Australia. A further five healthy participants (HC group) were recruited by advertising amongst community groups, hospital and university staff. The HC group did not have diabetes based on their medical history. The study took place between July and December 2012. The study was approved by The Townsville Hospital and Health Service and the James Cook University human research ethics committees (approval numbers HREC/12/QTHS/77 and H4693). Written informed consent was obtained from all participants prior to commencing the study.

### Training prior to reproducibility assessment

The researcher undertaking measurements in this study (MF) initially received extensive training from an expert in biomechanical assessments (RC). RC holds a PhD in biomechanics and is a trained exercise physiologist with more than 10 years’ experience in carrying out gait analyses. MF is a trained clinical podiatrist with 3 years clinical experience and limited prior experience in carrying out gait analyses. Approximately 40 h of customised training was provided by RC to MF prior to the assessments performed in this study. Training involved the placement of reflective markers on correctly identified anatomical landmarks [13], the accurate measurement of limb distances [15] and the accurate capturing and processing of plantar pressure and gait data based on pre-established protocols [10, 15–19]. The training was performed on 22 volunteers. All reproducibility assessments were carried out only after training had been completed to an adequate standard assessed by RC.

### Biomechanical assessments

One trained investigator (MF) conducted all assessments at the movement analysis laboratory at James Cook University, Townsville, Australia using a standard published protocol [14]. A range of approaches were used to assess the single-operator reproducibility of measurements utilising the pre-established protocol. Specifically, we examined the reproducibility of identifying key anatomical landmarks, measuring limb dimensions, processing three dimensional gait data and measuring plantar pressures as outlined below.

Height and weight were measured in all participants at the start of each session. These measurements were taken three times and averaged. Height was assessed using a wall mounted telescopic metal stadiometer (Seca model 220, Seca Scales, Hamburg, Germany). Body weight was measured using bioelectrical impedance scales (TANITA TBF 521, TANITA Corporation, and Arlington heights, Illinois, USA). Foot arch type was determined by a podiatrist (MF) by visually examining the participants arch while standing (weight bearing) and the arch was classified as either a pes planus foot type (flat foot), normal arch (neither flat foot or high arched foot) or as a pes cavus foot type (high arched foot).

### Identification of key anatomical landmarks

The correct placement of reflective markers on anatomical landmarks is a crucial initial step in obtaining reliable and valid gait data [11, 20]. We felt it important to firstly assess the degree of measurement error in placing reflective markers. We developed a simple method by which the reproducibility of identifying anatomical landmarks could be examined. This reliability assessment was carried out to assess if examiner one (MF) was able to correctly locate anatomical landmarks for the placement of reflective markers with precision when compared with examiner two, an expert in biomechanics (RC).

**Fig. 1:**
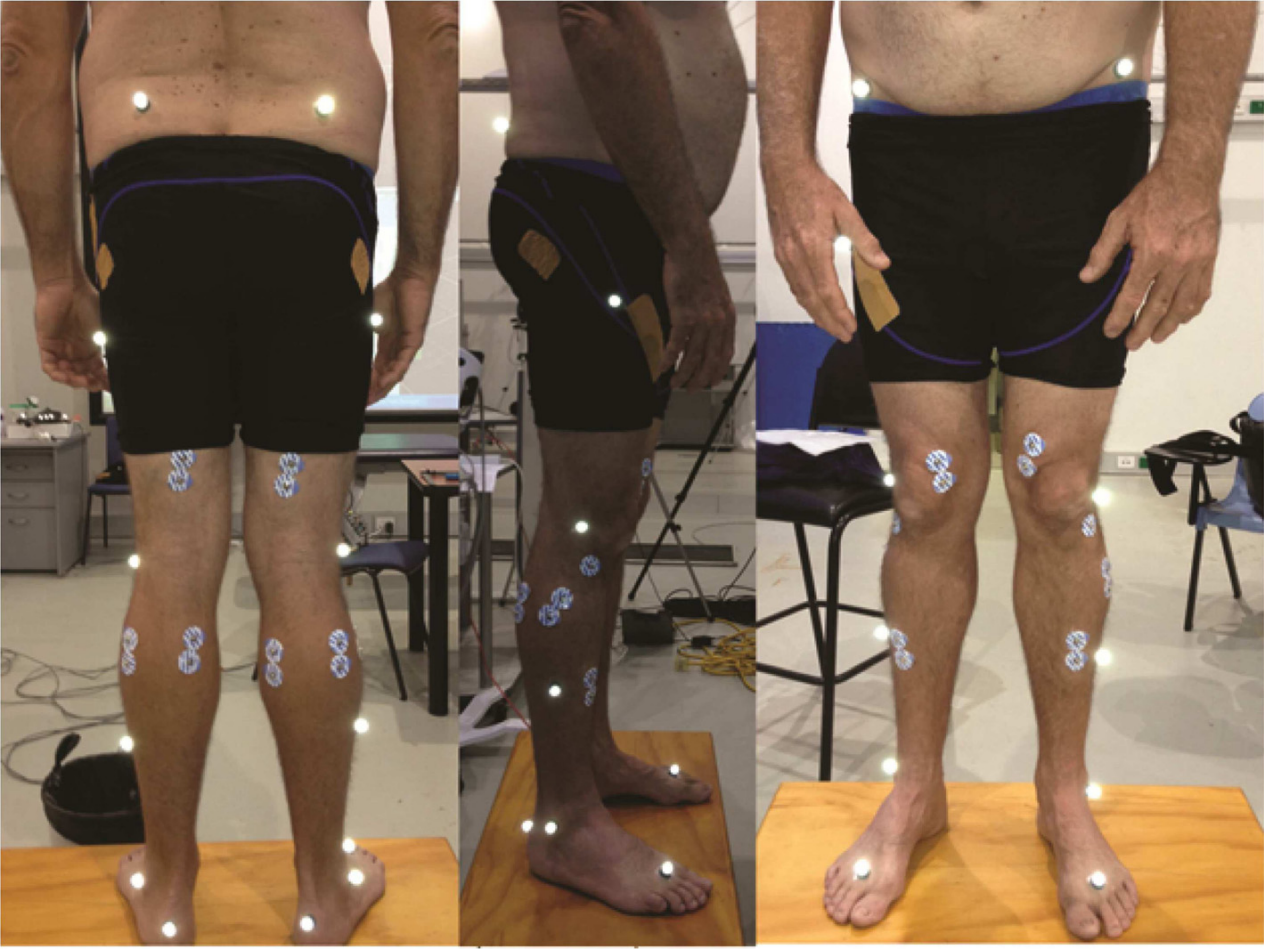
Anatomical locations used for reflective marker placement for the plug-in gait model. Legend: A participant standing in a relaxed stance position after reflective markers have been placed prior to three-dimensional movement analysis

Examiner one (MF) was asked to use a black coloured pen to identify and mark the exact positions of the anatomical locations utilised in the standard VICON Nexus plugin-gait analysis protocol [15]. This included 8 locations on both sides of the body (16 in total), following a Helen Hayes marker model [18]. These locations were the tibial tuberosity, head of fibula, lateral malleolus, medial malleolus, lateral shin (the mid-point of the leg between the knee and the ankle), the central posterior calcaneus, the head of second metatarsal and the anterior superior iliac spine (ASIS) (see Fig. 1). After examiner one (MF) had completed this task, examiner two (RC) was asked to place another pen marking of a different colour on where he perceived the anatomical landmarks to be. If examiner two believed that it was at the same location as examiner one, then no pen marking was inserted. After examiner two completed marking the anatomical locations, the difference between the two observers was measured in millimetres (mm).

A difference of more than seven mm was considered important as the base-diameter of the reflective markers used was seven mm. It was reasoned an error above this level in marker placement would likely impact on kinematic (movement) analyses outcomes.

### Measurements of leg dimensions utilised in constructing a three dimensional gait model

Accurate measurements of seven anatomical dimensions of the lower limb have to be performed when examining gait using the VICON Nexus plug-in gait model [15, 18]. These measurements and the reflective markers are used to acquire Euler angles during gait [18]. We performed measurements using a metal tape measure (KDSF10-02, KDS Corporation, Kyoto, Japan) and a handheld Vernier Caliper (Draper Tools Ltd., Hampshire, United Kingdom) in mm as previously described [15]. Anterior superior iliac spine (ASIS) width was defined as the distance from the left to the right ASIS (also known as the inter ASIS distance). Leg lengths were measured from each ASIS to the distal end of each medial malleolus (skeletal leg length). Knee diameter (knee width) was measured as the linear distance (medial to lateral) of the palpable knee joint margin while the knee was in full extension. Ankle diameter (ankle width) was taken as the distance from the anterior lateral edge of the lateral malleolus to the anterior medial edge of the medial malleolus while the participant was in the relaxed stance position (Fig. 1). Three measurements of leg dimensions were performed on each participant half an hour apart on the same day by the same examiner (MF).

### Processing of gait data

The movement analysis laboratory at James Cook University is equipped with the VICON gait analysis system (VICON, Oxford, United Kingdom). The system has ten T-40 series infrared cameras positioned around a walking environment capturing data at 100 Hz within the VICON Nexus movement analysis software (version 1.9.1, VICON, Oxford, United Kingdom). The laboratory also comprised of two 400 x 600 mm OR-6 AMTI force plates and two 900 x 900 mm OR-6 AMTI force plates (AMTI, Watertown, Massachusetts, USA) which were embedded on a 10 m long walking surface covered by concrete overlay. The force plates had a maximum excitation range of 10 V with each occupying six analogue channels (<2 % channel cross talk) which worked off a strain gauge bridge. The force plates were programmed to capture at a rate of 3000 Hz (3000 frames per second), for optimum capture speed whilst utilising all equipment simultaneously. All equipment was linked and synchronized with the VICON system in the laboratory. A similar system was used in a recent study investigating the gait of patients with trans-tibial amputation [21] and in another study assessing gait in patients with a history of foot ulcers [22].

**Fig. 2:**
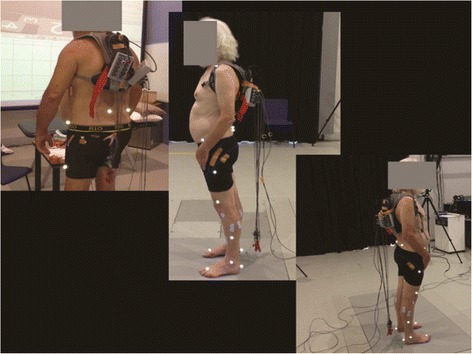
Participant getting ready to commence walking. Legend: A participant standing in the relaxed stance position, after reflective markers were attached. The figure indicates the positioning of the participant

A standard VICON Nexus procedure was used during motion capture (VICON Motion Systems, Oxford, England) [14, 15]. Participants were provided with tightly fitting shorts which conformed to the skin. This enabled the appropriate placement of reflective markers in the correct anatomical positions with minimal interference from clothing. Following marker placement, participants were instructed to position their feet against a ruler that was placed on the floor adjacent to the 10 m walking surface. Participants were instructed to keep their feet approximately shoulders length apart prior to walking. Walking commenced with the dominant leg. Participants were advised to practice walking in the assessment surrounding until they felt they had adopted their natural walking rhythm (see Fig. 2). We sought to establish the reliability of the manual processing of captured gait data. A single walking assessment was recorded from each participant. The same walking assessment was processed on ten occasions over five days (two per day) to establish the reproducibility in processing gait data. We did not examine the variability of gait in this study, but rather the reproducibility of analyzing gait data. The standard VICON pipeline procedure for processing gait data was used [15].

Temporal spatial parameters (TSPs) characterise various events, speeds and time intervals related to the gait cycle. We examined the variation in selected TSPs since they are imperative for calculating other important outcomes such as kinematics (angles) and kinetics (force) data. The TSPs included a total of 22 parameters measured in both legs, including walking speed, cadence, stride time, step time, opposite foot off time, opposite foot contact, foot off time, single support time, double support time, stride length and step length. These were calculated for the left and right limbs separately. The coefficient of variation (CV) was calculated for each TSP outcome related to each limb using the data outputs from ten processed extracts per participant. This was done by dividing each participant’s standard deviation (SD) by the mean and multiplying by 100. The average CV of the population was thus calculated from all individual CVs. The mean CV of each participant group was also determined. This provided a measurement of disagreement in the processing of gait assessments using standardised gait assessment software.

### Plantar pressures

The Footscan ® pressure plate (RSScan International, Olen, Belgium) was used for plantar pressure assessment with the associated Foot Scan ® processing software. This platform was 2000 mm in length, 400 mm in width and contained 16384 sensors capturing at 100 Hz. The plantar pressure platform was freestanding. The platform has been used for previous biomechanical research in participants that have diabetes [23] and has been confirmed as a reliable platform for plantar pressure measurements [23, 24]. The intention of this study was to assess the variation between measurements carried out on five consecutive days utilising a standard protocol [10, 19]. We employed the three step approach which involved the participant being trained to approach the plantar pressure platform so as to strike their third step (i.e. contact of the initiating limb) on the pressure platform first followed by the opposite foot second [19]. A reference marker was used to keep the starting point consistent for each walking assessment. All participants practiced walking over the platform several times until they were able to establish a comfortable pattern of walking over the platform. Instructions were repeated to obtain a gait pattern where the ipsilateral foot (initiating foot) would make contact with the platform on the third step as required [19].

Participants were asked to walk at a self-selected pace over the platform consecutively until ten walking assessments were acquired. A verbal cue was given to commence walking during each assessment. Participants were monitored to check that each assessment commenced at the reference marker placed on the floor. Participants were asked to ‘look straight ahead’ in order to prevent targeting foot placement on the platform. Data capture only commenced when a consistent gait pattern was achieved and the acquired pressure readings were visually consistent [14]. A consistent measurement was defined as a walking assessment in which all ten anatomical locations investigated were visible with a numerical pressure value recorded for each of the ten sites in both feet and where lateral or medial deviation of the foot off the pressure platform did not occur. The foot also had to be contained entirely within the active surface of the sensor array as detailed below [17]. Walking cadence was not adjusted or standardised and participants were allowed to adopt their natural walking pace [17]. A degree of intra-participant variability in walking and hence plantar pressures was anticipated due to normal variability in gait [25]. Prior research has suggested that adequate reproducibility can be obtained in measuring plantar pressure [26]. The variation between assessments of plantar pressures in healthy controls was reported to be 7 % for the lateral aspect of the rear foot and up to 20 % for the lateral forefoot for example [26].

We recorded fifty walking assessments per participant over five days. Five walking assessments were then randomly selected per day from the ten captured daily assessments per participant, as previously reported [16]. The selection of walking assessments for comparison was performed by one assessor (MF) who randomly selected two assessments from walks one to five, two assessments from walks six to ten and chose one further assessment from the remaining six walking assessments as the fifth included assessment per participant. Each plantar pressure assessment was only used once.

The pressure measurement software permitted masking of the foot to enable identification of plantar pressures at a total of 20 anatomical locations in both feet [17]. The locations included the plantar surfaces of the hallux, combined toes one to five, metatarsal one, metatarsal two, metatarsal three, metatarsal four, metatarsal five, the mid foot, the lateral rear foot and the medial rear foot. These measurements were reported by the software as the mean peak pressure (mpp), maximum sensor pressure (msp), pressure time integral (pti) and contact area (ca). When comparing the data, the mean and SD were first obtained for the mpp, msp, pti and ca for the ten plantar locations per individual over five days.

### Statistical analysis

The Statistical Package for the Social Sciences (SPSS) for Windows (released 2013, IBM SPSS Statistics for Windows, Version 22.0. Armonk, NY, IBM Corp) was used for the statistical analyses performed in this study. The differences between observers and 95 % confidence intervals (95 % CI) were computed to assess the accuracy of locating anatomical sites [27]. Lin’s concordance correlation coefficients (CCC) were calculated to examine the reproducibility of the three repeated measurements of leg dimensions [28]. These were reported with two sided 95 % CI using an online statistical program [29]. The CCCs were calculated by comparing the first and the second measurements, the first and the third measurements and the second and third measurements in all participants. We reported three CCCs to represent reproducibility between all there measurements as opposed to reporting an average CCC. CCCs were interpreted as almost perfect (>0.90), substantial (>0.8-0.9), moderate (0.65-0.8) and poor (<0.65) [30]. Bland-Altman plots were constructed for the assessments which had the lowest CCC values to illustrate the association between the mean difference in measurements and the mean leg dimension lengths [31]. CVs were used to assess the reproducibility of plantar pressure outcomes and TSPs. CVs are an accepted method of reproducibility evaluation in biomechanical data [25]. CVs were selected as there were far too many measurements to evaluate CCCs. The CVs were calculated by dividing the SD by the mean and multiplying by 100 to acquire a percentage (%). The CVs for the gait data were calculated by selecting one walking assessment from each participant and processing this assessment ten times over five days to obtain ten extracts of TSPs per participant. The individual CVs were calculated from the participant’s mean and SD. The mean CV of the population was then calculated by averaging the CVs of all individuals. For the TSPs, a CV of less than 10 % was defined as acceptable since it was reasoned that there should be a very low level of variation in the processing of gait data using standardised methods.

The CVs for mpp, msp, pti and ca were calculated by first acquiring a daily mean measurement for each participant from their five walks. The CVs in plantar pressure measurements (mpp, pti, msp and ca) over five days were calculated using the daily means and SDs from each participant. The mean CV of the population was subsequently calculated by averaging the CVs of all individuals. CV values for plantar pressure were considered to have good reproducibility if they were below 30 % as it was anticipated that readings between days would have a certain degree of inherent variability [26]. Continuous data were reported as median and interquartile range [IQR] and compared between groups using Kruskal-Wallis and Mann Whitney-U tests. Nominal data were presented as numbers and percentages (%) and compared between groups using Fisher’s exact test. We recruited participants from three distinct populations. Given the very different clinical characteristics of these participants, it was envisaged that the reproducibility might vary between groups. Thus the group specific reproducibility was investigated, despite the small sample sizes.

## Results

### Participant characteristics

The study population consisted of twenty eight limbs from fourteen participants (see Additional file 1 and Table 1 for a summary of characteristics).

**Table 1:** Participant characteristics

Variable	DFU group (*n* = 4)	DMC group (*n* = 5)	HC group (*n* = 5)	*p-value*
Age (yrs)	56.5 [47.0-71.3]	58.0 [52.0-64.0]	64.0 [52.0-72.5]	0.724
Diabetes duration (yrs)	17.0 [8.0-20.0]	3.0 [2.5-14.5]	-	0.138
Height (cm)	176.8 [165.5-179.1]	167.0 [159.5-168.5]	156.0 [154.8-172.5]	0.212
Weight (Kg)	128.5 [91.3-134.0]	79.4 [77.1-99.6]	73.0 [62.8-89.1]	0.061
Waist circumference (cm)	137.5 [113.0-140.3]	101.0 [91.0-119.0]	87.0 [80.0-98.50]	0.014
Hip circumference (cm)	129.5 [104.5-135.0]	98.0 [93.5-119.5]	89.0 [79.0-97.3]	0.034
Gender ratio [male: female]	3:1	2:3	2:3	0.080
Right foot arch type [pes planus: normal arch: pes cavus]	2:1:1	3:1:1	0:4:1	0.113
Left foot arch type [pes planus: normal arch: pes cavus]	2:1:1	3:1:1	0:4:1	0.113

Legend: The numerical values indicate the median and inter-quartile range. The *p*-values were derived from Kruskal Wallis test for between three group comparisons (DFU vs. DMC vs. HC) and the Mann–Whitney U test for two group comparisons (DFU vs. DMC group). Categorical variables were compared between groups using Fishers exact test. Diabetes duration indicates fractions of years diagnosed with type 2 diabetes mellitus

### The reproducibility of identifying anatomical landmarks

Table 2 displays the mean [95 % CI], minimum and maximum (absolute) differences in identifying all anatomical landmarks between observers for the entire study population. See Additional file 1 for a summary of results.

**Table 2:** Average differences between the novice and expert observers in the identification of anatomical sites in participants

Variable	Mean difference (mm)	SD of difference (mm)	Minimum difference (mm)	Maximumdifference (mm)	95 % confidence interval
Left Limb					
Tibial tuberosity	3	3	0	15	2-4
Head of fibula	2	2	0	6	2-3
Lateral malleolus	2	2	0	4	1-3
Medial malleolus	2	2	0	5	1-2
Lateral shin	1	2	0	5	0-2
Central posterior calcaneus	2	2	0	4	1-2
Head of second metatarsal	1	1	0	3	0-1
ASIS	5	4	0	10	3-7
Right limb					
Tibial tuberosity	3	3	0	10	2-4
Head of fibula	4	7	0	35	0-7
Lateral malleolus	2	2	0	6	2-3
Medial malleolus	2	2	0	5	1-2
Lateral shin	2	2	0	7	0-2
Central posterior calcaneus	2	2	0	6	1-3
Head of second metatarsal	1	1	0	3	0-1
ASIS	4	3	0	12	3-6

Legend Data relates to the difference between examiner 1 (MF) and expert examiner 2 (RC) in mm. A 7 mm difference was considered acceptable for this analysis. The SD relates to the standard deviation of the mean difference and the maximum and minimum values indicate the highest and lowest levels of measurement difference between the two observers. The two-sided 95 % confidence interval of the difference is also reported. ASIS = anterior superior iliac spine

### The reproducibility of assessing leg dimensions

**Fig. 3:**
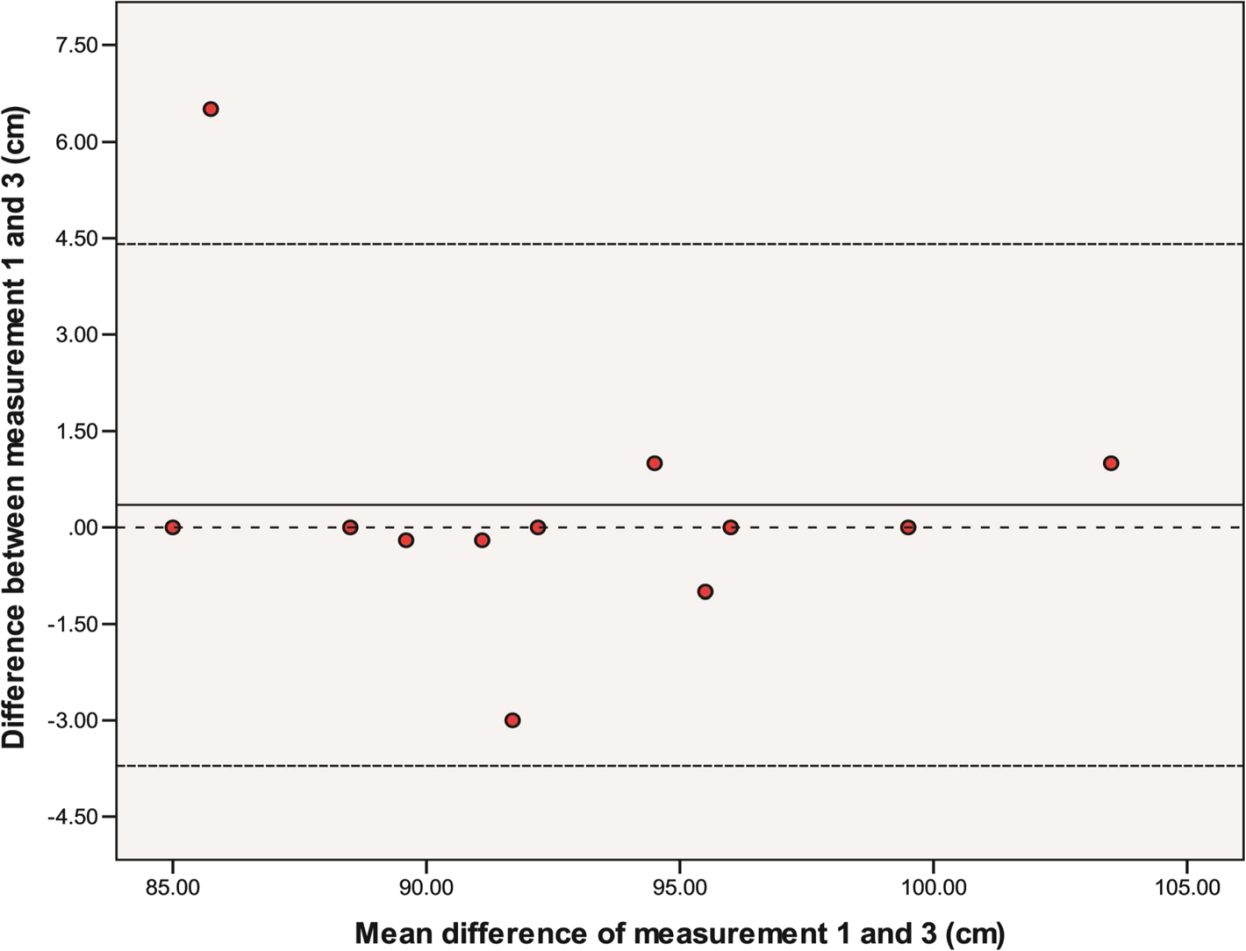
Bland Altman plot of left leg length [measurement one compared to measurement three]. Legend: A Bland and Altman plot of the difference between measurement one and measurement three for left leg length. The centre line of the graph indicates the mean difference between measurement one and three and the upper and lower dashed lines indicate the mean difference ± 2.00 times the standard deviation of the difference. The dashed centre line is the zero reference for the y axis. The y axis represents the difference of the two measurements and the x axis represents the mean of two measurements

The group CCC [95 % CI] values for limb and joint assessments for measurement 1 vs. 2; measurement 2 vs. 3 and measurement 1 vs. 3 are shown in Table 3. See Additional file 1 for a summary of results. The CCCs varied between 0.919 [95% CI 0.766-0.972] to 0.982 [95% CI 0.949-0.994] for left leg length and likely contained an outlier. See Fig. 3 for a Bland and Altman plot of the difference between measurement one and measurement three for left leg length.

**Table 3:** Concordance correlation statistics for the reproducibility of assessing leg dimensions on three occasions

Variable	CCC measurement 1 v 2 [95 % CI]	CCC measurement 1 v 3 [95 % CI]	CCC measurement 2 v 3 [95 % CI]
Left leg length	0.982 [0.949-0.994]	0.919 [0.776-0.972]	0.933 [0.822-0.975]
Left knee diameter	0.965 [0.895-0.988]	0.962 [0.891-0.987]	0.997 [0.990-0.999]
Left ankle diameter	0.961 [0.892-0.985]	0.944 [0.841-0.981]	0.971 [0.910-0.991]
Right leg length	0.994 [0.983-0.998]	0.972 [0.917-0.991]	0.977 [0.931-0.992]
Right knee diameter	0.986 [0.958-0.996]	0.984 [0.955-0.994]	0.980 [0.943-0.993]
Right ankle diameter	0.967 [0.902-0.989]	0.959 [0.878-0.986]	0.957 [0.876-0.985]
ASIS distance	0.994 [0.981-0.998]	0.995 [0.984-0.998]	0.997 [0.992-0.999]
Mass	0.999 [0.999-0.999]	0.999 [0.999-0.999]	0.999 [0.999-0.999]
Height	0.999 [0.999-0.999]	0.999 [0.999-0.999]	0.999 [0.999-0.999]

Legend: Concordance correlation coefficients (CCC) were calculated using http://www.niwa.co.nz/node/104318/concordance. Two sided 95 % confidence intervals are presented with the CCC value for each measurement. The strength of agreement was considered as: Almost perfect >0.90; Substantial >0.8-0.9; Moderate 0.65-0.8; and Poor <0.65. ASIS = anterior superior iliac spine

### The reproducibility of processing gait measurements

The mean CVs for the repeated processing of gait data were all considerably below the acceptable 10 % (Table 4). See Additional file 1 for a summary of results.

**Table 4:** Mean coefficient of variation for gait measurements in the study population

Variable	Left limb CV (%)	Right limb CV (%)
Cadence	0.1	0.1
Walking speed	0.5	0.6
Stride time	0.2	0.3
Step time	0.5	0.5
Opposite foot off	0.7	0.8
Opposite foot contact	0.1	0.1
Foot off time	0.1	0.2
Single support time	0.8	0.8
Double support time	1.3	1.9
Stride length	0.4	0.6
Step length	0.6	0.9

Legend: CV = Coefficient of variation reported as a percentage. Cadence refers to number of steps per minute. The two measurements reported as <0.1 contained CVs which were below 0.001. A pre-established level of <10 % was used as a threshold for determining appropriate variance in gait measures

### The reproducibility of plantar pressure measurements

The calculated mean CVs for plantar pressures are presented in Table 5. See Additional file 1 for a summary of results.

**Table 5:** Mean coefficient of variation for plantar pressure measurements in the study population

Variable	mpp CV (%)	pti CV (%)	ca CV (%)	msp CV (%)	Variable	mpp CV (%)	pti CV (%)	ca CV (%)	msp CV (%)
Left foot					Right Foot				
*Toe1*	32.1	41.3	26.2	32.7	*Toe1*	24.5	31.2	18.2	32.3
*Toes 2-5*	31.5	38.7	31.5	39.6	*Toes 2-5*	25.9	44.1	28.5	32.7
*Metatarsal 1*	27.9	34.9	16.2	35.0	*Metatarsal 1*	25.9	26.3	16.2	28.7
*Metatarsal 2*	22.5	22.0	11.2	31.0	*Metatarsal 2*	20.9	21.1	14.5	24.4
*Metatarsal 3*	21.4	22.1	9.5	30.5	*Metatarsal 3*	18.3	19.1	13.4	20.6
*Metatarsal 4*	26.1	28.9	10.3	35.3	*Metatarsal 4*	19.3	21.0	10.5	20.4
*Metatarsal 5*	31.1	35.7	19.8	41.2	*Metatarsal 5*	20.4	22.9	11.8	21.5
*Midfoot*	21.8	25.6	16.3	27.4	*Midfoot*	13.7	17.1	7.6	21.4
*Medial Heel*	19.1	23.1	7.2	24.4	*Medial Heel*	19.3	24.4	7.8	26.5
*Lateral Heel*	21.4	22.0	8.0	25.2	*Lateral Heel*	19.3	25.0	7.9	26.3

Legend: Mean peak pressure (mpp), pressure time integral (pti), contact area (ca) and maximum sensor pressure (msp) in a plantar anatomical location. CV = Coefficient of variation reported as a percentage. A pre-established level of <30 % was used as a threshold for determining appropriate reproducibility

### Influence of group on the reproducibility of measurements

See Additional file 1 for a summary of results and Additional file 2 for group data.

## Discussion

This study aimed to describe the reproducibility of one operator in performing key measurements needed for three dimensional gait and plantar pressure assessments using standard protocols. This study was felt to be an important prerequisite prior to undertaking a longitudinal study investigating the changes in gait and plantar pressures during foot ulcer healing in a cohort of participants with type 2 diabetes [7, 11, 12]. Overall, the findings suggest that following extensive training, a junior operator can obtain the necessary skills to accurately identify key anatomical landmarks and measure leg dimensions needed for gait analyses. The reproducibility of gait measurements and plantar pressure measurements were mostly within the pre-defined levels of acceptability. The levels of acceptable reproducibility used were set at arbitrary thresholds and are open to criticism. In reference to plantar pressure, the mpp measurement was the most reliable, followed by the pti. Variation in plantar pressure was worse in the left limb. The reasons for this need further clarification.

The assessment of biomechanical parameters requires the reliable identification of anatomical landmarks [11, 20]. One of the methodological challenges lies in the difficulty of placing anatomical markers in precise locations during testing sessions. Errors in marker placement may alter the orientation of body segments, leading to errors in kinematic curves created during gait [7]. We assessed the competency of a trained examiner in consistently assessing the locations for reflective marker placement when compared to an expert in the field of biomechanics. Our results suggest that the difference between assessors was acceptable for all anatomical landmarks. The mean differences and the 95 % CIs for almost all landmarks were less than seven mm, which we defined as an important difference. We hypothesised that if the base of the marker measuring 7 mm (the attachment point on the skin) was incorrectly placed outside of an anatomical location then it is likely that errors in measurements would result. The maximum differences at a few anatomical sites were however greater than seven mm. Assessments performed at the right head of the fibula was an example where the upper limit of the 95 % confidence interval of the mean difference was above the pre-determined limit. This error was due to the large maximum difference noted in one female participant in the HC group with genu recurvatum and a posteriorly displaced head of fibula. Differences in marker placement of greater than 25 mm have previously been suggested to be important when examining spine movements, although it is likely smaller differences may impact lower limb assessments [32].

A recent study supports our finding that a novice with previous clinical experience is able to learn the operation of three dimensional gait analyses with good reliability compared to an expert in the field [13]. Our participants and methods of reproducibility assessment however differed considerably from this recent study [13]. Leigh and colleagues used a much younger cohort of healthy, lean participants and utilised post-processing kinematic data from treadmill walking to assess measurement reproducibility. This method of assessing reproducibility has the potential to be significantly influenced by the natural gait variability of participants which can increase the intra-participant variability level [11]. While Leigh and colleagues concentrated on reflective marker placement, we investigated reflective maker placement and additional parameters such as limb distance measurements and gait data processing to encompass a number of steps that can lead to measurement errors in gait assessments [13]. Leigh and colleagues reported that the inter-observer reproducibility of marker placement was good based on coefficients of correlations between assessors for a number of kinematic measurements exceeding 0.9 [13]. The findings from our study, when combined with the findings of Leigh and colleagues, suggest that gait can be assessed reproducibly when standard protocols are used which observers are familiar with.

Accurate assessment of anatomical distances within the lower limbs is required to construct models used in gait assessment. We therefore examined the reproducibility of one observer assessing these distances on three occasions. The CCC statistics for repeat assessment of limb dimensions were within an acceptable level of agreement based on our pre-established criteria [28]. The lowest CCC was found in the measurement of left knee diameter at 0.644 [95 % CI 0.212-0.940] in the HC group. We felt these results still reflected an acceptable level of agreement [30]. We also believe this finding reflected an outlier, likely resulting from a recording-error rather than a measurement error. When this outlier was removed the CCC value increased. We are not aware of any other studies which have assessed repeated measurements of anatomical distances used in gait analysis. We did not examine the reproducibility of kinematic outcomes which would have required comparing multiple gait assessments that contain natural intra-participant gait variability [26]. Our assessment suggested good reproducibility of TSPs when a standard protocol was used by one operator to process gait data. The highest CV was for double support time, which was above 1 %. We considered this level of operator processing error to be insignificant as such a small variation in processing TSPs would have a minor impact on the final outcomes of other kinematic and kinetic variables.

There are nevertheless several other methods by which error can be introduced during gait assessment which were not assessed in this study [11, 33]. These include errors associated with instrumentation, errors caused by the poor placement of cameras, the size of the area and volume being assessed by the cameras and errors caused by the incorrect calibration of cameras before data capture [33]. We nonetheless believe that most of these errors should be small when a single trained assessor is conducting measurements based on a standardised protocol [11].

The CV values for the assessment of plantar pressures suggested that the measurement of msp had the highest variability while mpp, pti and ca had lower overall variability. Our findings suggested greater variation in mpp, pti, ca and msp readings in the DFU group and the HC group compared to the DMC group. The range of CVs in our HC group (4 to 49 %) showed greater variability than previously reported (7 to 20 %) [26] which may be because we performed a larger number of measurements. A study by Bacarin and colleagues previously reported CVs of approximately 40 % and 50 % for pti and mpp assessments, respectively, in participants with a history of DFUs [25]. The range of CVs in our DFU group was lower (13.9 to 38.9 % for pti and 13.2 to 31.5 % for mpp). We defined a CV of <30 % as being reasonably reproducible as this was within the range previously reported [25, 26]. Although the CVs for most plantar pressure variables were below our pre-established level, several measurements were also higher than the proposed threshold. It is established that a considerable degree of natural variation occurs during gait and thus in plantar pressure in most healthy participants [34]. Hence this finding was not unexpected in this heterogeneous sample.

Variability in the assessment of plantar pressure could be due to a number of factors. These include variation in participant foot placement and error in the location assignment for plantar sites across walking measurements [17, 35]. The high CVs in plantar pressures that were observed in our DFU group may represent variations in gait due to severe peripheral neuropathy and the presence of DFUs [6]. Our findings are similar to those previously reported [25]. The reasons for this observed variability requires further investigation using a larger cohort. Although a distinctly higher level of variation in plantar pressure measures were noted in the left foot of participants, the cause of this is difficult to ascertain given the small sample size and the design of this study. This result may be related to limb dominance as a majority of our participants were right-limb dominant.

Wearing and colleagues (1999) reported that there were three main factors to be considered when acquiring plantar pressures: (1) the level of reliability required; (2) the foot site being assessed; and (3) the type of pressure measurement studied (for example mpp as opposed to pti) [36]. By using the third step from the commencement of walking (i.e. the three step method) and five walking assessments, it was found that reliable data could be obtained [36]. The overall reliability of any variable, however, appeared to be site-dependent [36]. It is important to recall that there is natural variation in gait and it is currently unknown how this variation in gait is important in healthy populations and in disease. Our study highlights that when studying populations with foot pathology such as those with plantar foot ulcers, the reproducibility of plantar pressure assessments may be reduced due to natural gait variation and that these measurements are foot and site dependent [36]. On average, the reproducibility of plantar pressure was poorest when measured in the forefoot and toe areas compared to the heel and the mid foot. This finding was similar to that of Wearing and colleagues [36]. As the CVs for mpp, pti and ca measurements were the highest at the first toe and the lesser toes, it is highly likely that a smaller surface area in these particular regions may have impacted on the increased variation in plantar pressure. Although the plantar pressure platform contained 16384 sensors which were capturing at 100 Hz, this may still be insufficient to accurately assess plantar pressure in small areas such as the tips of toes. This is a technical limitation and highlights the importance of reporting plantar pressure outcomes according to each individual site, rather than combining plantar pressures or averaging plantar pressures for a given foot region as has been the previous practice. It is also possible that variation in the placement of the participants’ feet on the pressure platform contributed to this finding. The sensor density and sensor number utilised to record each individual pressure measurement may also have influenced these results. The mpp was found to be the most reproducible out of the three plantar pressure measures and is likely the most suitable plantar pressure outcome for the population of interest. We believe that using a three step protocol and assessing plantar pressure according to its appropriate plantar site and as three distinct measurements are important requirements, especially when evaluating patients with plantar foot ulcers [37]. Nevertheless, the reported CVs still need to be considered as a limitation in assessing plantar pressures longitudinally. CVs may be reduced by providing adequate practice time for participants and by perhaps increasing the number of steps captured [36].

This study has a number of limitations. The sample was small and heterogeneous, although it represented the populations of interest. The number of individuals in the different groups was particularly small and likely affected our ability to compare reproducibility between recruitment groups effectively. The reason for the small sample size was the need for detailed assessments on each participant and the requirement for multiple visits. This limited the ability to recruit a large sample. We attempted to recruit a group of participants that best represented the population who are commonly examined by the techniques assessed. The participants recruited were also representative of those to be investigated in a future prospective cohort study [14]. We based our level of acceptability in plantar pressure readings on variances reported in previously published studies [25, 26] and hypothesised an acceptable level of variability for gait trial processing. The arbitrary cut-offs we used as acceptable levels of reproducibility need to be further validated as these have not been investigated previously. The study would have benefitted by blinding assessors or by using a larger number of assessors, particularly in evaluating the reproducibility of identifying anatomical locations. Despite these limitations, we believe the observations outlined in this small study may be useful when considering measurement reproducibility in gait and plantar pressure studies in populations who have known foot ailments. The findings may be useful for others planning to establish biomechanical testing in similar populations.

## Conclusion

The current study reports on the reproducibility of several key measurements needed to examine gait and plantar pressures. Overall, the findings suggest that these measurements can be reliably assessed by a trained observer using currently available standardised protocols. We believe investigating the reproducibility of methods is important prior to initiating longitudinal collection of gait and plantar pressure data and for later comparison of results.

## Availability of supporting data

The data set supporting the results of this article are available in the [ResearchOnline@JCU] repository, [unique persistent identifier 2044–6055 https://researchonline.jcu.edu.au].

## Additional files

Additional file 1:
**Containing a summary of the results of the study.** (DOCX 15 kb) Additional file 2:
**Containing additional data from subgroup analyses.** (DOCX 29 kb)

## Abbreviations

DPN: diabetic peripheral neuropathy
DFU: diabetic foot ulcer
DMC: diabetes mellitus control
HC: healthy control
ASIS: anterior superior iliac spine
TSP: temporal spatial parameters
mpp: mean peak pressure
msp: maximum sensor pressure
pti: pressure time integral
ca: Contact area
CCC: concordance correlation coefficient
CV: coefficient of variation
