# Supplementary material for: The reproducibility of acquiring three dimensional gait and plantar pressure data using established protocols in participants with and without type 2 diabetes and foot ulcers
Source: J Foot Ankle Res. 2016 Jan 29;9:4. doi: 10.1186/s13047-016-0135-8 (PMC4731914; doi:10.1186/s13047-016-0135-8)
Supplement: Additional file 1: — Containing a summary of the results of the study. (DOCX 15 kb) [file 13047_2016_135_MOESM1_ESM.docx]

**Supplementary File 1: Results**

**Participant characteristics**

There were three males and one female in the DFU group; two males and three females in the DMC group; and two males and three females in the HC group (see Table 1). The median age [IQR] was 56.5 [47.0-71.3] in the DFU group; 58.0 [52.0-64.0] in the DMC group and 64.0 [52.0-72.5] in the HC group. There was no statistical difference in age, gender, foot arch-type, diabetes duration, height or weight between groups (*p*>0.05). The hip circumference and waist circumference however differed between groups. The DFU group had a larger hip and waist circumference compared to the other two groups (see Table 1).

**The reproducibility of identifying anatomical landmarks**

Table 2 displays the mean [95% CI], minimum and maximum (absolute) differences in identifying all anatomical landmarks between observers for the entire study population. Almost all mean differences and 95% CIs were within the pre-defined 7mm level of agreement, except the 95% CI for the right head of the fibula which was between 0 and 7 mm. The maximum group difference was greater than the pre-defined 7mm for five of the 16 landmarks assessed, namely the left tibial tuberosity (15 mm), the left ASIS (10 mm), the right tibial tuberosity (10 mm), the right head of fibula (35 mm) and the right ASIS (12 mm).

**The reproducibility of assessing leg dimensions**

The group CCC [95% CI] values for limb and joint assessments for measurement 1 vs. 2; measurement 2 vs. 3 and measurement 1 vs. 3 are shown in Table 3. The CCCs between the three measurements ranged between 0.919 [95% CI 0.766-0.972] to 0.982 [95% CI 0.949-0.994] for left leg length and likely contained an outlier (see Figure 3). Removing the outlier from one participant increased the lowest CCC value to 0.976 [95% CI 0.928-0.992]. The range of CCCs for other leg dimensions were 0.944 [95% CI 0.841-0.981] to 0.997 [95% CI 0.990-0.999] for the left leg and between 0.957 [95% CI 0.876-0.985] to 0.980 [95% CI 0.943-0.993] for the right leg.

**The reproducibility of processing gait measurements**

The mean CVs for the repeated processing of gait data were all considerably below the acceptable 10% (Table 4). The highest CVs were noted for double support-time in both left and right limbs (1.3% and 1.9% respectively). All other CV values were below 1% (Table 4).

**The reproducibility of plantar pressure measurements**

The calculated mean CVs for plantar pressures are presented in Table 5. The right foot demonstrated a smaller range of CVs compared to the left foot. Contact area demonstrated the highest reproducibility over five days for both feet (CVs <30% in 19/20 locations) and was followed by mpp (CVs <30% in 17/20 locations) and pti (CVs <30% in 14/20 locations. CVs for msp demonstrated the lowest level of reproducibility overall. In the left foot, only 3/10 CVs for msp were <30% compared to 8/10 CVs for the right foot (see Table 5). The highest CVs for mpp and pti and ca measurements were at toe 1 for the left foot and toes 2-5 for the right foot. The highest CVs for msp were at metatarsal 5 in the left foot (41.2%) and toes 2-5 in the right foot (32.7%).

**Influence of group on the reproducibility of measurements**

We found no clear association between participant group and the reproducibility of assessing anatomical locations and leg dimensions (Supplementary Tables 1 and 2). Large CVs in assessing double support time were observed within all three groups (see Supplementary Table 3). The CVs for plantar pressure measurements appeared to vary little between groups and were generally low for all groups for msp and pti (see supplementary Table 4). Out of all the plantar pressure outcomes, the msp measurements had the higher CVs in all groups. The CVs for mpp measurements were lower than the pti and msp measurements in all groups. The DFU group had 59/80 CVs which were <30%, the DMC group had 68/80 CVs which were <30% and the HC group had 58/80 CVs which were <30% (see Supplementary Table 4). The left foot measurements for mpp, pti, ca and msp had much higher CVs compared to the right foot irrespective of group (see Supplementary table 4).
