# Supplementary material for: The reproducibility of acquiring three dimensional gait and plantar pressure data using established protocols in participants with and without type 2 diabetes and foot ulcers
Source: J Foot Ankle Res. 2016 Jan 29;9:4. doi: 10.1186/s13047-016-0135-8 (PMC4731914; doi:10.1186/s13047-016-0135-8)
Supplement: Additional file 2: — Containing additional data from subgroup analyses. (DOCX 29 kb) [file 13047_2016_135_MOESM2_ESM.docx]

**Supplementary File 2: Supplementary Tables with group specific data**

**Supplementary Table 1: Assessment of reproducibility by sub-groups for anatomical measurements**

| **Variable** | **DFU (n=4)** | **DMC (n=5)** | **HC (n=5)** |
| --- | --- | --- | --- |
| **Left Limb** |  |  |  |
| Tibial tuberosity | 4 | 4 | 4 |
| Head of fibula | 0 | 4 | 4 |
| Lateral malleolus | 4 | 4 | 3 |
| Medial malleolus | 5 | 4 | 4 |
| Lateral shin | 4 | 0 | 5 |
| Central posterior calcaneus | 3 | 3 | 4 |
| Head of second metatarsal | 3 | 3 | 3 |
| ASIS* | 10 | 10 | 3 |
| **Right limb** |  |  |  |
| Tibial tuberosity | 5 | 6 | 0 |
| Head of fibula | 4 | 3 | 35 |
| Lateral malleolus | 6 | 3 |  |
| Medial malleolus | 5 | 4 | 4 |
| Lateral shin | 3 | 2 | 2 |
| Central posterior calcaneus | 4 | 3 | 6 |
| Head of second metatarsal | 3 | 0 | 3 |
| ASIS | 5 | 12 | 0 |
|  |  |  |  |

Legend: The numerical value indicates the maximum difference in (mm) for measurements between two assessors by group. Difference relates to the difference between examiner 1 (MF) and examiner 2 (RC). For purposes of clinical significance a 7mm margin of error was acceptable for this analysis. *ASIS = anterior superior iliac spine. A zero indicates measurements for which differences did not exist between the two assessors.

**Supplementary Table 2: Assessment of reproducibility by sub-groups for limb measurements**

| **Variable** | **CCC measurement 1 v 2 [95% CI]** | **CCC measurement 1 v 3 [95% CI]** | **CCC measurement 2 v 3 [95% CI]** |
| --- | --- | --- | --- |
| **DFU group (n=4)** | | | |
| Left leg length | 0.992[0.947-0.998] | 0.987[0.887-0.998] | 0.998[0.970-0.999] |
| Left knee diameter | 0.951[0.554-0.995] | 0.941[0.551-0.993] | 0.991[0.906-0.999] |
| Left ankle diameter | 0.961[0.762-0.994] | 0.938[0.906-0.960] | 0.960[0.791-0.993] |
| Right leg length | 0.997[0.965-0.999] | 0.992[0.901-0.999] | 0.995[0.952-0.999] |
| Right knee diameter | 0.972[0.789-0.996] | 0.982[0.820-0.998] | 0.964[0.610-0.997] |
| Right ankle diameter | 0.831[0.108-0.986] | 0.961[0.532-0.997] | 0.920[0.419-0.991] |
| ASIS distance | 0.999 [0.999-0.999] | 0.999[0.999-0.999] | 0.999[0.999-0.999] |
| Mass | 0.999[0.995-0.999] | 0.999[0.996-999] | 0.999[0.995-0.999] |
| Height | 0.999[0.998-0.999] | 0.999[0.996-0.999] | 0.999[0.999-0.999] |
| **DMC group (n=5)** | | | |
| Left leg length | 0.920[0.474-0.990] | 0.704[0.030-0.945] | 0.774[0.252-0.960] |
| Left knee diameter | 0.997[0.986-0.999] | 0.996[0.967-0.999] | 0.997[0.987-0.999] |
| Left ankle diameter | 0.927[0.609-0.988] | 0.944[0.692-0.991] | 0.995[0.956-0.999] |
| Right leg length | 0.979[0.909-0.995] | 0.915[0.632-0.982] | 0.929[0.564-0.990] |
| Right knee diameter | 0.965[0.866-0.991] | 0.957[0.783-0.992] | 0.992[0.929-0.999] |
| Right ankle diameter | 0.992[0.982-0.996] | 0.989[0.921-0.998] | 0.989[0.927-0.998] |
| ASIS* distance | 0.988[0.899-0.998] | 0.993[0.941-0.999] | 0.997[0.977-0.999] |
| Mass | 0.998[0.998-0.999] | 0.999[0.997-0.999] | 0.999[0.998-0.999] |
| Height | 0.999[0.999-0.999] | 0.999[0.998-0.999] | 0.999[0.997-0.999] |
| **HC group (n=5)** | | | |
| Left leg length | 0.997[0.982-0.999] | 0.992[0.933-0.999] | 0.997[0.976-0.999] |
| Left knee diameter | 0.644[0.212-0.940] | 0.657[0.220-0.946] | 0.996[0.982-0.999] |
| Left ankle diameter | 0.978[0.939-0.992] | 0.855[0.329-0.976] | 0.899[0.374-0.987] |
| Right leg length | 0.996[0.974-0.999] | 0.987[0.899-0.998] | 0.973[0.810-0.996] |
| Right knee diameter | 0.996[0.981-0.999] | 0.995[0.964-0.999] | 0.973[0.810-0.996] |
| Right ankle diameter | 0.936[0.599-0.991] | 0.758[0.07-0.968] | 0.812[0.05-0.976] |
| ASIS distance | 0.994[0.939-0.999] | 0.993[0.942-0.999] | 0.996[0.982-0.999] |
| Mass | 0.999[0.999-0.999] | 0.999[0.999-0.999] | 0.999[0.998-0.999] |
| Height | 0.998[0.988-0.999] | 0.999[0.998-0.999] | 0.998[0.989-0.999] |
|  |  |  |  |

Legend: Numerical figures indicate CCC measurements and two-sided 95% confidence intervals. Concordance Correlation Coefficients (CCC) were calculated using http://www.niwa.co.nz/node/104318/concordance. Two sided 95% confidence intervals are presented with the CCC value for each measurement. The strength of agreement was considered as: Almost perfect >0.90; Substantial >0.8-0.9; Moderate 0.65-0.8; and Poor <0.65. *ASIS = anterior superior iliac spine.

**Supplementary Table 3: Coefficients of variation (CV) for repeated processing of gait trials**

| **Variable** | **DFU group (n=4) CV (%)** | **DMC group (n=5) CV (%)** | **HC group (n=5) CV (%)** |
| --- | --- | --- | --- |
| **Left limb** |  | | |
| Cadence | 0.1 | 0.2 | 0.0 |
| Walking speed | 0.7 | 0.3 | 0.6 |
| Stride time | 0.1 | 0.2 | 0.2 |
| Step time | 0.9 | 0.3 | 0.3 |
| Opposite foot off | 0.3 | 0.5 | 1.1 |
| Opposite foot contact | 0.1 | 0.2 | 0.0 |
| Foot off time | 0.1 | 0.1 | 0.1 |
| Single support time | 1.5 | 0.4 | 0.8 |
| Double support time | 1.8 | 0.6 | 1.7 |
| Stride length | 0.6 | 0.2 | 0.5 |
| Step length | 1.1 | 0.2 | 0.7 |
| **Right limb** |  | | |
| Cadence | 0.0 | 0.2 | 0.2 |
| Walking speed | 0.6 | 0.3 | 0.8 |
| Stride time | 0.6 | 0.2 | 0.2 |
| Step time | 1.1 | 0.2 | 0.4 |
| Opposite foot off | 0.2 | 1.1 | 1.0 |
| Opposite foot contact | 0.1 | 0.2 | 0.0 |
| Foot off time | 0.0 | 0.2 | 0.2 |
| Single support time | 1.6 | 0.4 | 0.6 |
| Double support time | 1.6 | 2.1 | 1.9 |
| Stride length | 0.5 | 0.6 | 0.7 |
| Step length | 1.2 | 1.0 | 0.6 |
|  |  |  |  |

Legend: Figures represent coefficients of variation (CV) reported as percentages (%).Cadence refers to number of steps per minute. The CV measurements reported as 0.0 contained CVs which were below 0.001.

**Supplementary Table 4: Coefficients of variation (CV) for plantar pressure measurements by group for left and right foot.**

| **Variable** | **DFU group (n=4) CV (%)** | | | | **DMC group (n=5) CV (%)** | | | | **HC group (n=5) CV (%)** | | | |
| --- | --- | --- | --- | --- | --- | --- | --- | --- | --- | --- | --- | --- |
|  | *MPP* | *PTI* | *CA* | *MSP* | *MPP* | *PTI* | *CA* | *MSP* | *MPP* | *PTI* | *CA* | *MSP* |
| **Right foot** |  | | | | | | | | | | | |
| Toe1 | 21.5 | 30.4 | 12.0 | 27.3 | 18.9 | 23.3 | 12.0 | 29.5 | 32.5 | 39.7 | 29.3 | 39.2 |
| Toes 2-5 | 31.4 | 38.0 | 22.8 | 23.7 | 25.2 | 44.1 | 27.2 | 35.9 | 22.2 | 48.9 | 34.3 | 36.6 |
| Metatarsal 1 | 30.9 | 30.4 | 24.4 | 27.5 | 23.4 | 24.4 | 11.6 | 27.9 | 24.4 | 24.9 | 14.3 | 30.5 |
| Metatarsal 2 | 29.8 | 29.9 | 24.0 | 22.0 | 14.4 | 17.1 | 14.0 | 23.5 | 20.4 | 17.9 | 7.5 | 27.1 |
| Metatarsal 3 | 25.6 | 29.4 | 22.4 | 18.7 | 12.4 | 14.3 | 12.8 | 19.4 | 18.3 | 15.6 | 6.8 | 23.3 |
| Metatarsal 4 | 25.3 | 29.1 | 18.1 | 25.2 | 16.2 | 17.6 | 8.3 | 15.4 | 17.7 | 17.9 | 6.4 | 21.4 |
| Metatarsal 5 | 27.4 | 32.1 | 17.6 | 25.8 | 18.3 | 18.9 | 9.8 | 19.7 | 16.8 | 19.5 | 9.3 | 19.8 |
| Midfoot | 13.2 | 18.2 | 8.1 | 18.0 | 14.3 | 17.6 | 5.6 | 27.3 | 13.5 | 15.9 | 9.2 | 18.3 |
| Medial Heel | 15.6 | 35.1 | 15.6 | 19.2 | 16.9 | 19.7 | 5.1 | 22.9 | 24.6 | 20.5 | 4.3 | 36.0 |
| Lateral Heel | 21.0 | 37.2 | 16.3 | 21.3 | 20.1 | 24.3 | 4.5 | 32.3 | 17.1 | 16.1 | 4.6 | 24.3 |
| **Left foot** |  | | | | | | | | | | | |
| Toe1 | 26.0 | 38.0 | 38.9 | 27.4 | 30.5 | 36.9 | 17.2 | 34.8 | 38.5 | 48.3 | 25.0 | 34.9 |
| Toes 2-5 | 31.5 | 38.0 | 36.8 | 38.9 | 29.1 | 37.6 | 26.4 | 38.5 | 33.7 | 40.5 | 32.5 | 41.3 |
| Metatarsal 1 | 29.5 | 38.9 | 15.1 | 32.7 | 26.1 | 29.5 | 12.7 | 36.5 | 28.6 | 36.9 | 20.7 | 35.5 |
| Metatarsal 2 | 14.3 | 13.9 | 7.5 | 23.7 | 21.9 | 22.9 | 7.7 | 28.4 | 29.6 | 27.5 | 17.8 | 39.5 |
| Metatarsal 3 | 16.7 | 17.8 | 6.6 | 31.5 | 23.3 | 23.2 | 6.7 | 30.7 | 23.4 | 24.3 | 14.5 | 29.6 |
| Metatarsal 4 | 23.7 | 27.1 | 6.5 | 38.6 | 24.3 | 25.8 | 7.1 | 27.4 | 30.0 | 33.4 | 16.3 | 40.6 |
| Metatarsal 5 | 28.8 | 38.0 | 18.6 | 40.2 | 29.2 | 31.0 | 16.9 | 36.6 | 35.0 | 38.4 | 23.8 | 46.5 |
| Midfoot | 28.3 | 36.3 | 13.9 | 28.5 | 22.1 | 23.9 | 6.2 | 25.1 | 16.2 | 18.7 | 28.4 | 28.8 |
| Medial Heel | 23.6 | 28.8 | 4.0 | 23.8 | 16.4 | 21.3 | 4.3 | 22.6 | 18.4 | 20.5 | 12.6 | 26.6 |
| Lateral Heel | 20.5 | 24.0 | 5.1 | 15.3 | 19.2 | 16.2 | 4.9 | 25.5 | 24.4 | 26.1 | 13.4 | 32.9 |
|  |  |  |  |  |  |  |  |  |  |  |  |  |

Legend: Figures represent the average coefficients of variation (CV) reported as percentages (%).Maximum sensor pressure in a plantar anatomical location (msp), mean peak pressure in a plantar anatomical location (mpp), pressure time integral (pti) and contact area (ca). CV= Coefficient of variation reported as a percentage for each group based on the average CV of the group from five selected walks per individual over five days.
